# Diseases of the Respiratory System

**Published:** 1904-10-01

**Authors:** 


### DISEASES OF THE RESPIRATORY SYSTEM.

**Abscess of the Lungs.**—This condition, when not due to tuberculosis, may be caused by pneumonia, foreign bodies, trauma, embolism, perforation of neighbouring ulcers and bronchiectasis, according to Schlesinger.[1] Physical signs of cavity and the characteristic sputum, especially if the former varies with the expectoration of the latter, are the chief diagnostic signs. The Roentgen rays are valuable in diagnosis, there is also an increasing leucocytosis, and in cases without expectoration increasing dulness in normally resonant lung is an important sign. Pleural adhesions are very common, sucking in of the intercostal spaces and œdema over the site of the abscess may occur. Holt[2] points out that the diagnosis usually has to be made from unresolved pneumonia, sacculated empyema, and tuberculosis. The differentiation is sometimes impossible, but he states that the fever is usually higher in abcess and the leucocytosis more marked than in simple unresolved pneumonia. Loud coarse pleuritic râles are unusual in sacculated empyema, and moreover the pus is more easily reached by the exploring needle. Abscess most usually follows pneumonia, and this with the history may distinguish it from tuberculosis, as may the absence of wasting and the occurrence of pneumococci in the pus withdrawn. Holt's two cases which occurred in young children were both successfully treated by surgical means, as was a case following septic pneumonia reported by Sharp and Keeling.[3] This case, which occurred in an adult woman, apparently arose from the inspiration of foul material from the mouth (carious teeth), and was cured by simple aspiration. A resection of the ribs subsequently performed showed that no infection of the pleura by the aspirating needle had taken place. Schlesinger states that 80 per cent. of abscesses recover after operation, but only 60 per cent. of cases of gangrene. If the abscess is not foul the operation may be put off for a few weeks. He also notes that operation for bronchiectasis is very unsatisfactory.

**Adrenalin.**—This has been used with success by Hedley[4] in two cases of pulmonary hæmorrhage in phthisis, 1 in 5,000 supra-renalin solution (Armour) being administered in drachm doses three times a day. Duncanson,[5] however, considers that adrenalin is dangerous in hæmoptysis, and describes a case in which a considerable rise of blood-pressure apparently took place under its use. He attributes this to the effect of the drug which has been shown experimentally to cause general contraction of the blood-vessels, except in the pulmonary area, where they are dilated. Barr[6] for the last two years has been injecting a solution of adrenalin locally in cases of serous effusion. For the treatment of pleuritic effusion he uses one drachm of Parke, Davis and Co.'s adrenalin chloride, 1 in 1,000. In addition he now injects sterilised air to prevent the formation of pleural adhesions. For this purpose a special apparatus has been devised. Barr considers that the treatment is eminently successful, though unnecessary in mild cases, where the fluid is rapidly absorbed if left to itself. He insists on the importance of careful aseptic precautions, and does not recommend the treatment where the fluid is even slightly purulent.

**Pleural Effusion.**—In the differential diagnosis between simple and purulent effusion Buchanan[7] does not consider Bacelli's sign of great value. He thinks that pus often gives physical signs more closely resembling consolidation, while serum has a selective, rather than an inhibitive, influence on vocal vibrations. Gullan[8] describes a case in which tactile vocal fremitus and the voice and breath sounds were transmitted through a purulent effusion, and the general symptoms were attributed to advanced tuberculous lesions which were actually present at the apices. Warrington[9] does not consider that the nature of the effusion influences the character of the breath sounds. The diagnosis between pleural effusion and solid lung may be made by the obliquity of the upper limit of the line of dulness (Warrington), the altered position of the apex beat, and the extreme dulness with a sense of resistance (Armstrong).[10] Davies attaches importance to the loss of vocal fremitus but advises exploratory puncture in doubtful cases.

**Injuries to the Chest Wall.**—Three cases have been described by James[11] in which severe fits of coughing resulted in fracture of the ribs, in two of which, however, the diagnosis could not be established with absolute certainty. The cases all occurred in adults of middle age. He has also collected 40 cases from the literature and believes the condition more common than it is usually thought to be, many cases of localised and severe thoracic pain being possibly caused in this manner. Walker[12] thinks actual fracture rare, but mentions that separation of the epiphysis may occur as the result of slight force. Bryant[13] considers that the diaphragm and serratus magnus are the muscles capable of causing fracture of the ribs, and owing to the presence of the liver on the right side, this would be more likely to happen on the left. The cases showed that fracture was more frequent on the left side, and intercostal neuralgia was also said to be commoner on this side. Hence the thought that some cases of the latter might in reality be instances of fracture, especially as the callus was likely to be very small. The false motion should help in the diagnosis where fracture was present, and also the ease and rapidity with which the condition could be cured.

1 Lancet, Feb. 6, 1904, p. 403. 2 Arch. of Pediat., 1904, vol. xxi. 3 Lancet, May 7, 1904, p. 1278. 4 Brit. Med. Jour., Feb. 13, 1904. p. 365. 5 Ibid., March 12, 1904, p. 603. 6 Ibid., March 19, 1904, p. 649. 7 Ibid., May 7, 1904, p. 1080. 8 Ibid. 9 Ibid. 10 Ibid. 11 Med. Rec., Dec. 5, 1903, p. 917. 12 Ibid. 13 Ibid.

*(To be continued.)*

